# Genomics and Physiology of a Marine Flavobacterium Encoding a Proteorhodopsin and a Xanthorhodopsin-Like Protein

**DOI:** 10.1371/journal.pone.0057487

**Published:** 2013-03-04

**Authors:** Thomas Riedel, Laura Gómez-Consarnau, Jürgen Tomasch, Madeleine Martin, Michael Jarek, José M. González, Stefan Spring, Meike Rohlfs, Thorsten Brinkhoff, Heribert Cypionka, Markus Göker, Anne Fiebig, Johannes Klein, Alexander Goesmann, Jed A. Fuhrman, Irene Wagner-Döbler

**Affiliations:** 1 Helmholtz-Centre for Infection Research, Braunschweig, Germany; 2 University of Southern California, Department of Biological Sciences, Los Angeles, California, United States of America; 3 University of La Laguna, Department of Microbiology, La Laguna, Tenerife, Spain; 4 Leibniz Institute DSMZ – German Collection of Microorganisms and Cell Cultures, Braunschweig, Germany; 5 Carl von Ossietzky University, Institute of Chemistry and Biology of the Marine Environment (ICBM), Oldenburg, Germany; 6 University of Braunschweig, Department of Microbiology, Braunschweig, Germany; 7 Center for Biotechnology, University of Bielefeld, Bielefeld, Germany; Wageningen University, The Netherlands

## Abstract

Proteorhodopsin (PR) photoheterotrophy in the marine flavobacterium *Dokdonia* sp. PRO95 has previously been investigated, showing no growth stimulation in the light at intermediate carbon concentrations. Here we report the genome sequence of strain PRO95 and compare it to two other PR encoding *Dokdonia* genomes: that of strain 4H-3-7-5 which shows the most similar genome, and that of strain MED134 which grows better in the light under oligotrophic conditions. Our genome analysis revealed that the PRO95 genome as well as the 4H-3-7-5 genome encode a protein related to xanthorhodopsins. The genomic environment and phylogenetic distribution of this gene suggest that it may have frequently been recruited by lateral gene transfer. Expression analyses by RT-PCR and direct mRNA-sequencing showed that both rhodopsins and the complete β-carotene pathway necessary for retinal production are transcribed in PRO95. Proton translocation measurements showed enhanced proton pump activity in response to light, supporting that one or both rhodopsins are functional. Genomic information and carbon source respiration data were used to develop a defined cultivation medium for PRO95, but reproducible growth always required small amounts of yeast extract. Although PRO95 contains and expresses two rhodopsin genes, light did not stimulate its growth as determined by cell numbers in a nutrient poor seawater medium that mimics its natural environment, confirming previous experiments at intermediate carbon concentrations. Starvation or stress conditions might be needed to observe the physiological effect of light induced energy acquisition.

## Introduction

Rhodopsins are photoactive membrane-embedded opsins found in all three domains of life [1]. These energy transduction proteins contain seven-transmembrane α-helices and use retinal as a cofactor, located in the retinal-binding pocket, for their light-driven function. Microbial rhodopsins, classified as type I rhodopsins, are widespread amongst prokaryotes and can function as proton pumps [bacteriorhodopsins (BR) and proteorhodopsins (PR)], ion pumps [(halorhodopsins (HR)] and photosensors [sensory rhodopsins (SRI and SRII)]. BR was discovered in *Halobacterium salinarium*, an extremely halophilic archaeon, found in hypersaline lakes and salterns [2], [3]. Bacterial rhodopsin (proteorhodopsin, PR) was discovered much later in a metagenome fragment of a marine gammaproteobacterium from the SAR86 clade [4], [5]. More than a decade after their discovery, research on PRs has shown their extensive abundance and diversity in aquatic ecosystems suggesting a key role in the biochemical cycles of the oceans.

Xanthorhodopsins (XRs) are light-harvesting proton pumps with a dual chromophore, one retinal molecule and one carotenoid antenna, that were first discovered in *Salinibacter ruber* [6]. Its carotenoid antenna salinixanthin transfers as much as 40–45% of the absorbed photons to retinal [7], resulting in a potentially much more efficient light-capturing system compared to PRs from Bacteria [4], [5] or BRs from Archaea [2]. XRs have a binding pocket into which both retinal and a second chromophore, e.g. salinixanthin (*S. ruber* [6] or echinenone (*Gloeobacter violaceus* [8]) are positioned.

Environmental metagenomics and single cell genomics have shown PRs to be abundant not only in the ocean [9], [10], [10]–[18], but also in freshwater bacterioplankton [19], where they were found for the first time in *Deltaproteobacteria*, *Verrucomicrobia* and *Sphingobacteria* [20]. In their study, the authors also obtained evidence for lateral gene transfer: Actinobacterial PRs were found in several gammaproteobacterial genomes; moreover, in one actinobacterial genome, two PR genes were present, one related to *Actinobacteria,* the other one typical for *Betaproteobacteria*. Sequencing the genomes of single PR-carrying bacteria recovered by cell sorting from the ocean showed that their genomes were more representative of the natural microbial bacterioplankton community than those of cultivated PR-containing bacteria [21]. However, although this new approach has a great potential to reveal the presence of certain genes in single representative genomes, it does not show if the gene is expressed and what is its physiological role for the organism that is carrying it. Experiments using heterologous systems, on the other hand, can show the function of the encoded protein. They demonstrated the proton pump activity of PRs found in the environment [4] and showed that the proton gradient might be used to power flagella movement [22]. The question puzzling marine microbiologists, however, remains unanswered by those approaches, namely, what is powered in marine microbes by the rhodopsins that many of them are carrying? Only culturing of a strain that naturally contains the gene has the potential to explain how this organism uses the rhodopsin to survive in its particular habitat.

A large percentage of whole genome sequenced PR-containing bacteria belong to the family *Flavobacteriaceae*. However, and despite the genomic similarities, the consequences of light exposure on the different flavobacterial species appear to vary. Whereas light stimulated growth in *Dokdonia* sp. MED134 [23], [24], no light-induced growth advantage was observed for its relative *Dokdonia* sp. PRO95 [25]. No growth advantage in the light was also reported for the alphaproteobacterium *Candidatus* Pelagibacter ubique HTCC1062 [26], the flavobacterium *Polaribacter* sp. MED152 [27], and the gammaproteobacteria *Vibrio* sp. AND4 [28] and *Vibrio campellii* BAA-1116 [29]. However, light exposure seems to activate other metabolic processes. In *Polaribacter* sp. MED152 light increases the amount of bicarbonate fixed by anaplerotic enzymes [27]. In *Candidatus* Pelagibacter ubique HTCC1062 light induces ATP production for endogenous carbon respiration under starvation conditions [30]. For *Vibrio* sp. AND4 light was shown to increase long-term survival during starvation in seawater [28]. Similarly, in *Vibrio campellii* BAA-1116 the presence of light resulted in increased survival during stress [29]. Taken together, those studies suggest that the genomic context of the different bacteria might play an important role for defining the physiological consequences of PR-phototrophy. However, why strains of the same species present different physiological responses remains unresolved. Thus, the reason for the distinct light responses in *Dokdonia* sp. MED134 and *Dokdonia* sp. PRO95 which are 99.8% similar on the level of the 16S rRNA gene could rely on genome variations apparently unrelated to the PR gene itself.

Here we report the genome sequence of strain *Dokdonia* sp. PRO95 and compare it to that of *Dokdonia* sp. MED134 and *Dokdonia* sp. H4-3-7-5 (formerly *Krokinobacter* sp. 4H-3-7-5 [31], [32]). We analyzed the presence of genomic islands, differences in gene content, and sigma factor-dependent regulation. The discovery of a second rhodopsin in PRO95 prompted us to investigate its genomic context, phylogenetic affiliation, and transcription in more depth. Guided by genome information, we conducted cultivation experiments and attempted to find a defined medium for PRO95. We performed physiological experiments to determine light-induced proton pumping activity. The effect of light on growth of PRO95 was studied in seawater culture, i.e. at extremely low nutrient concentrations similar to those found in the natural environment.

## Materials and Methods

### Sampling and Isolation of Strain PRO95

Strain PRO95 was isolated from a surface water sample collected at the “Kabeltonne” at Helgoland Roads, a sampling site between the two islands of Helgoland (Germany), 60 km offshore in the North Sea (54° 11.18′ North, 7° 54′ East). No specific permits were required for the described field study. The sampling site is not privately owned or protected in any way. The sampling of water did not involve endangered or protected species. The sampling and isolation procedure has already been described [25]. *Dokdonia* sp. PRO95 has been deposited at the DSMZ (DSM 26625).

### Cultivation of Strain PRO95

Strain PRO95 was cultivated on plates of marine agar 2216 (Difco, Becton, Dickinson and Company, Heidelberg, Germany) at room temperature (20–25°C) for two days. For liquid cultures, first precultures were made. Single colonies were transferred to marine broth 2216 (Difco, Becton, Dickinson and Company, Heidelberg, Germany) which was diluted as required. Dilutions were made either with distilled water, in which case the media were amended with Sea salts (Sigma-Aldrich, St. Louis, USA) to obtain the salt concentration of full strength marine broth 2216 (31.8 g per liter) as described previously [25], or with natural sterile filtered autoclaved seawater from the North Sea, in order to change only the concentration of nutrients, but not the salt concentration. Precultures and main cultures were incubated at 25°C and 160 rpm in a natural day-light cycle. For DNA extraction, cells were cultivated in full strength marine broth 2216.

For RNA extraction cells were cultured in half strength marine broth 2216 amended with Sea salts and in 250 times diluted marine broth 2216 medium diluted with natural seawater.

For proton translocation measurements cells were cultivated overnight at room temperature and 150 rpm in 8× diluted marine broth 2216 medium amended with Sea salts (0.75 g nutrients per liter, 30.25 mM C).

### DNA Extraction

Genomic DNA was isolated using the NucleoSpin® Tissue Kit (Macherey & Nagel, Düren, Germany) according to the manufacturer’s instructions. For cell lysis, samples were incubated with proteinase K overnight. Afterwards a RNase digestion was performed to obtain RNA-free genomic DNA. The purity of the DNA was controlled by gel electrophoresis and by using a NanoDrop 1000 spectrophotometer (PEQLAB Biotechnology GmbH, Erlangen, Germany) as well as the Qubit 2.0 Fluorometer (Life Technologies, Darmstadt, Germany).

### Genome Sequencing and Assembly

For genome sequencing two libraries were prepared: A fosmid library with the CopyControl Fosmid library Production Kit containing the PCC1FOS Vector (EPICENTRE Biotechnologies, Madison, USA) was constructed according the manufacturer’s instructions. Moreover, a DNA library of 300 bp was prepared according to the manufacturer’s instructions “Preparing Samples for Paired-End-Sequencing”. Briefly, the genomic DNA was fragmented using a nebulization technique and the Covaris S2 system (Covaris Inc., Woburn, USA). Afterwards the overhangs resulting from DNA-fragmentation were converted into blunt ends. Additionally A-bases were added to the 3′-end of the blunt phosphorylated DNA fragments for the following adapter-ligation to the ends of the DNA fragments. Ligation products were gel-purified and an appropriate size-range of templates was selected for the cluster generation platform. DNA fragments were hybridized onto the flow-cell by the Illumina Cluster Station and were amplified for sequencing on the Genome Analyzer IIx (Illumina Inc., San Diego, USA). Clustered template DNA was sequenced using a robust four-color DNA Sequencing-By-Sequencing (SBS) technology. DNA fragments were sequenced for 76 bp (*de novo*-sequencing*)* and 150 bp (fosmid-sequencing) paired end. The fluorescent images were processed using the Illumina Genome Analyzer Pipeline Analysis Software 1.8. The sequencing outcome is summarized in Table S1. DNA sequences were assembled with Velvet 0.7.51 [33]. The assembly was controlled manually using the Staden Package Release 1-7-1b [34]. Misassemblies were eliminated using Gap4 (Staden package) and Hawkeye (AMOS 3.0.1 tool collection). Residual gaps were closed with PCR using specific primers. PCR products were purified with the PCR Clean-Up & Gel-Extraction Kit (SLG-Südlabor, Gauting, Germany) and sequenced on the Genome Analyzer IIx (Illumina Inc., San Diego, USA). Received sequences were manually controlled and further assembled with the whole genome sequence as described above. Genes were automatically annotated on the GenDB platform [35] followed by a manual annotation of genes of interest. The genome sequence of *Dokdonia* sp. PRO95 was submitted to Genbank (Accession No. ANPJ00000000; PID PRJNA176625).

### Lateral Gene Transfer Analysis

In order to detect lateral gene transfer (LGT) the normalized tetranucleotide frequency was calculated for the whole genome and sliding windows of 5 kb or 10 kb with 2.5 kb or 5 kb overlap, respectively using the formula
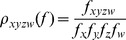
where 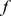 denotes the DNA sequence, 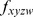 is the frequency of tetranucleotide 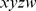 and 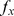 to 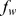 are the corresponding mononucleotide frequencies. Similarity of local and global tetranucleotide frequencies was calculated as Pearson correlation coefficient. Several studies demonstrated the reliability of this indicator to identify LGT [36], [37]. The threshold for counting a region as laterally transferred was calculated based on the interquartile-range of the correlation coefficients



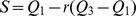
where 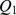 and 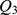 are the first and third quartiles, respectively. The value of 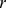 was set to 2: Average amino acid identity (AAI) was calculated as reciprocal best hits (RBH) between each pair of genomes (coverage> = 50, identity> = 30, evalue< = 1) as described [38]. For assignment of BLASTP hits to the NCBI taxonomy, the program MEGAN 4 [39] was used. Genomic islands specific for PRO95 were defined as regions with a tetranucleotide correlation coefficient below the threshold value and genes missing in both related *Dokdonia* strains. In cases were reciprocal best hits (RBHs) were found with correlation coefficients below the cutoff, the corresponding regions might represent LGT specific to *Dokdonia*. When RBH were missing in regions with no atypical tetranucleotide frequency, gene loss in the other *Dokdonia* strains was assumed.

### Phylogenetic Analysis of Rhodopsin Protein Sequences

The rhodopsin amino acid sequence tree was created using ClustalW for sequence alignment and the neighbor-joining method for tree construction using the software MEGA 5.03 [40]–[42]. Evolutionary distances were calculated by the Poisson correction method and are expressed as numbers of amino acid substitutions per site. Positions corresponding to alignment gaps were eliminated. Bootstrap sampling (1000 replicates) was used for validation.

### Genomic Environment Analysis of Both Rhodopsins

Genes for rhodopsins, its genomic environment, and retinal synthesis were identified using the GenDB annotation platform and NCBI BLAST analysis [43]. A comparison was performed with the reference genomes listed in Information S2. The lengths of ORFs and non-coding regions were manually retrieved and calculated.

### Extraction of Total RNA

RNA was isolated using the RNeasy Mini Kit (Qiagen, Hilden, Germany). In addition to the on-column DNase I treatment using the RNase-free DNase I Set (Qiagen, Hilden, Germany), a second DNase I digestion was performed in solution. The RNA concentration was determined using a NanoDrop 1000 spectrophotometer (PEQLAB Biotechnology GmbH, Erlangen, Germany), and its quality was evaluated using a 2100 Bioanalyzer (Agilent, Santa Clara, CA, USA).

### Amplification of Total RNA

0.5 µg of the extracted total RNA were used for amplification using the MessageAmpII-Bacteria Kit (Ambion). Briefly, total RNA was polyadenylated using *E. coli* poly(A) polymerase. The poly(A)-tails were reverse transcribed (Array Script reverse transcriptase) primed with an oligo(dT) primer bearing a T7 promotor. The double-stranded cDNA was transcribed *in vitro* at 37°C for 14 h, resulting in large amounts of single-stranded antisense RNA.

### Removal of Ribosomal RNA (rRNA) using Terminator 5′ Phosphate-dependent Exonuclease

16S and 23S rRNA was removed from total amplified antisense RNA using Terminator 5′ Phosphate-dependent Exonuclease (TDE, EPICENTRE, Madison, USA). A sample of 20 µg of amplified RNA was mixed with 10× TDE reaction buffer, 9U TDE, and sterile diethyl pyrocarbonate-treated water to obtain a final volume of 30 µl. The reaction mix was incubated for 2.5 h at 30°C in a thermocycler. To stop the reaction a termination by phenol extraction and ethanol precipitation was performed. To the reaction mix RNase-free water was added to obtain a total volume of 150 µl. The phenol extraction was performed by adding 400 µl buffer saturated phenol (P:C:I) and vortexing for 30 s. The reaction mix was centrifuged for 15 min at room temperature. The aqueous phase was transferred to a new vial and 0.1 volume of 3 M sodium acetate and 2.5 volumes of ethanol (70%) were added. The reaction mix was incubated at −20°C for 30 min. RNA centrifugation was performed at full speed (15000×g) for 30 min at 4°C, the supernatant was discarded and the RNA pellet was washed two times with 500 µl of 70% ethanol (and centrifuged each time for 5 min at 7.500 rpm and 4°C). The resulting RNA pellet was air-dried and resuspended in 30 µl RNase-free water.

### Removal of Ribosomal RNA (rRNA) using MicobExpress Bacterial mRNA Enrichment kit

Ribosomal RNA (16S and 23S) was removed using the MicrobExpress Bacterial mRNA Enrichment Kit (Ambion Inc., Austin, USA). The manufacturer’s protocol was performed using 5 µg of total RNA. In brief: After heating the total RNA and the Capture Oligonucleotide Mix to 70°C for 10 min, the hybridization was performed at 37°C for 15 min. Oligo MagBeads were prepared and heated to 37°C in binding buffer. Afterwards they were added to the RNA/Capture OligoMix and were incubated at 37°C for 15 min. The remaining mRNA was recovered and precipitated at −20°C overnight in a mix of 1/10^th^ volume of 3 M sodium acetate, 1/50^th^ volume of glycogen and 3 volumes of 100% ethanol. After centrifugation (13000 rpm, 4°C, 30 min) the pellet was washed two times with 70% ethanol. The resulting pellet was air dried and resuspended in nuclease-free water (Sigma-Aldrich, St. Louis, USA). Finally residual MagBeads were removed using a MagnaRack (Invitrogen, Carlsbad, USA) again. The MicrobExpress Kit was used for the experiment with 1/2 MB) and the Terminator 5′-Phosphate-dependent Exonuclease was used for the experiment with 1/250× diluted marine broth medium 2216.

### Illumina Solexa RNA Sequencing

The RNA samples were sequenced using 36 bp single end sequencing on a Genome Analyzer IIx (Illumina). The fluorescent images were processed using the Genome Analyzer Pipeline Analysis software 1.8 (Illumina). The sequence output was transformed to FastQ format, mapped against the genome sequence of PRO95 and the expression levels were calculated using the RNA-Seq tool of CLC Genomics Workbench V.4.7.2 software. Testing for a linear relationship between samples was performed in the R-Environment using the glm (generalized linear models) function.

### Seawater Culture Experiments

For seawater culture an artificial seawater medium (Sea Salts, Sigma-Aldrich, St. Louis, USA; 35 psu of salinity) was filter-sterilized and autoclaved. The medium was supplemented with 0.14 mM C by adding the appropriate amount of MB 2216. To avoid inorganic nutrient limitation, final concentrations of 2.1 µM NH_4_Cl and 0.3 µM Na_2_HPO_4_ [23] were added. To avoid impurities, all used materials were rinsed with 1 M HCl and washed with MilliQ water. As inoculum for the main cultures, precultures previously grown in seawater in a natural day-night cycle were used. Main cultures were incubated in polycarbonate bottles (Nalgene, Rochester, USA) in the light or in constant darkness (covered with black bags). For bacterial counts, samples were fixed in formalaldehyde (4% final concentration), filtered onto a black-stained 0.22 µm polycarbonate filter (Millipore, Billerica, USA), stained with Acridine Orange and counted by epiflourescence microscopy.

### Proton Translocation Measurements

750 ml of freshly grown cells were harvested by centrifugation (10 min, 8.000 rpm, 4°C). The cell pellet was washed immediately with 100 ml artificial seawater, resuspended and centrifuged again. This washing step was repeated once more. Afterwards the washed cell pellet was resuspended in a final amount of 5 ml of artificial seawater. Proton translocation was analysed by measurement of pH (Inlab423 electrode, Mettler-Toledo, Giessen, Germany) using oxygen saturated artificial seawater pulses that were added to the PRO95 cell suspensions in a glass reaction chamber containing a magnetic stirrer that was closed with an appropriate rubber plug. For light-induced proton pump analysis, LED spotlights (Osram Decospot) emitting white light (0.84 W, ∼ 95 µEinstein s^−1^ m^−2^, measured by a LI-250A Light Meter, LI-COR Biosciences, Lincoln, USA) were used. Harvested and washed cells of PRO95 were suspended in artificial seawater supplemented with 500 mM KSCN [44]–[47]. Proton translocation measurements were performed under anoxic condition (permanent addition of nitrogen). For calibration of the pH measurement system small pulses of 1 mM anoxic HCl were added in a known concentration of 50 nmol. The pH signal was measured in mV. For data acquisition a 12 bit A/D converter (LabJackU12, Meilhaus Electronic, Puchheim, Germany) together with the MPwin software written by H. Cypionka (http://www.pmbio.icbm.de/download/MPwinU12.zip) was used.

As negative control for proton translocation 25 µM TCS diluted in methanol wasadded to the PRO95 cell suspension. To eliminate the artefact of light-induced reactions of pH-electrodes, the used pH electrode was also tested for reaction only in cell-free medium.

### Phenotype Microarrays

For characterization of carbon source metabolism in PRO95 the Omnilog system (Biolog platform) with phenotype microarray plates PM1 and PM2 was used according to the manufacturer’s instructions. Experiments with both PM plates were performed in two independent replicates, which showed the same results. To allow quantitative comparison of results, the area under curve (AUC) was determined as a measure of respiratory activity for each tested carbon source using Simpson’s rule. Respiratory activity was assumed to be positive for an AUC that was 50% higher than that of the negative control.

### PCR Amplification and Sequencing

Primers used in this study are shown in Table S3. PCRs were performed as described previously [25], except using the Hot Master Mix 2.5× (5 PRIME, Hamburg, Germany) and the indicated annealing temperatures. PCR products were purified with the PCR Clean-Up & Gel-Extraction Kit (SLG-Südlabor, Gauting, Germany) and visualized by gel electrophoresis. The identity of the amplified fragments was confirmed by sequencing. For simultaneous PCR amplification of both rhodopsin genes, degenerated primers were designed with MEGA v5.03 coupled with ClustalW using default parameters for multiple alignments and the Gonnet protein weight matrix. A total volume of 50 µl was used containing ∼10 ng of template and 1 µM of each primer. An annealing temperature of 58°C was used.

RT-PCRs were performed as described [25] in a final volume of 20 µl except that an annealing temperature of 58°C and 59°C was used. RT-PCR products (PR 158 bp, XR-like 159 bp) were purified with the PCR Clean-Up & Gel-Extraction Kit (SLG-Südlabor, Gauting, Germany) and visualized by gel electrophoresis. DNA-fragments were further directly sequenced. Nucleotide sequences of the forward and reverse read were assembled.

## Results

### Genome Analysis and Comparison

#### Gene content comparison

**Figure 1:**
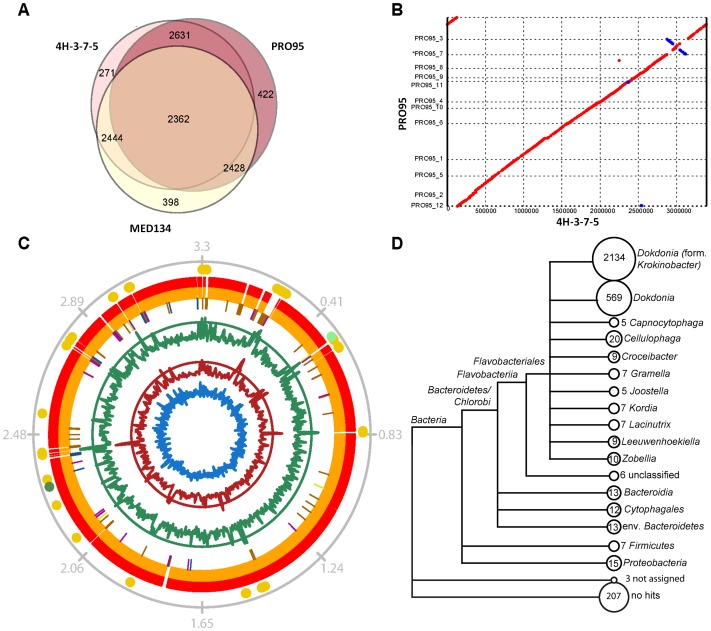
Comparative Genomics of *Dokdonia* sp. PRO95. A: Venn-Diagram comparing the genomic content of three *Dokdonia* species. B: Synteny-plot comparing *Dokdonia* sp. PRO95 and 4-H-3-7-5. C: Detection of lateral gene transfer (LGT) in the genome of PRO95. From outside to inside: sequence address in nucleotides (grey); identified genomic islands (yellow) and position of xanthorhodopsin (light green) and proteorhodpsin gene (green); reciprocal best hits (RBHs) in *Dokdonia* sp. 4H-3-7-5 (red); and *Dokdonia* sp. MED134 (orange); RBHs in flavobacteria (brown), *Bacteroidetes* (green), *Proteobacteria* (blue) and *Firmicutes* (purple); tetranucleotide frequency correlation and cut –off line, 5 kb window (green); tetranucleotide frequency correlation and cut –off line, 10 kb window (red); GC content (blue). D: Phylogenetic distribution of best BLASTP hits of all PRO95 ORFs. Organisms delivering best hits were assigned to taxonomic groups according to the NCBI taxonomy.

Table 1 shows genome statistics for the three closely related strains *Dokdonia* sp. PRO95, *Dokdonia.* sp. MED134, and *Dokdonia* sp. 4H-3-7-5. The genome size of 4H3-7-5 is slightly larger than that of MED134 and PRO95 (3.4 vs. 3.3 Mbp), probably because the genome of 4H3-7-5 is finished while the two other genomes consist of up to 12 fragments. In agreement with the 16S rRNA tree, the GC content of MED134 is higher than that of PRO95 and 4H-3-7-5. Accordingly, AAI between PRO95 and 4H-3-7-5 is higher (92.71%) than between PRO95 and MED134 (84.11%) or between MED134 and 4H-3-7-5 (82.15%). The closer similarity between PRO95 and 4H-3-7-5 is also shown in the Venn diagram (Fig. 1A). Mummer was used to align the genome of PRO95 against 4H-3-7-5 and Fig. 1B shows that PRO95 aligns almost completely with 4H-3-7-5.

**Table 1:** General features of the three genomes.

General features	MED134	PRO95	4H375
nt	3,301,953	3,303,993	3,389,993
GC content (%)	38.2	37.4	37.3
Coding density (%)	90.4	90.7	91.1
Predicted protein coding genes	3008	3111	2978
tRNA’s	43	36	42
Genes in paralogous clusters	417	443	468
Lipopeptides	238	231	257
Proteins with signal peptide	392	342	382
Peptidases	106	100	104
Glycosyl hydrolases	17	13	15
Adhesion proteins	39	30	33
Transporter genes	137	136	146
ABC transporters	45	49	49
TonB transporters	27	30	26

Data are based on the manual annotation of MED134, GenDB annotation in the case of PRO95, and 4H-3-7-5 GenBank file. Peptidases, glycosyl hydrolases, adhesion proteins and transporter genes were recalculated as the number of hits to the corresponding PFAMs, and the genes in paralogous clusters were calculated as described [28]. Lipoproteins were identified using LipoP (http://www.ncbi.nlm.nih.gov/pubmed/12876315) and signal peptides with SignalP (http://www.ncbi.nlm.nih.gov/pubmed/21959131).

With respect to functional classes of proteins, all three strains are very similar. Amongst the most abundant classes of predicted peptides, PRO95 has 100 peptidases, MED134 has 106 and 4H-3-7-5 has 104. This is a high number compared with most bacteria. *Dokdonia* strains probably degrade proteins, e.g. gelatin as suggested for MED134 [48]. PRO95 has 13 glycosyl hydrolases, MED134 has 17 and 4H-3-7-5 has 15. Although these numbers are also relatively high, the three strains seem to have a strong preference for peptides over polysaccharides. Peptides with adhesion domains are involved in the degradation of high molecular weight compounds. PRO95 has 30, MED134 has 39 and 4H-3-7-5 has 33, indicating that all three strains are likely to carry out a lifestyle attached to particles and are adapted to growth on high molecular weight compounds, e.g. proteins. Additionally environmental sequences closely related to the genus *Dokdonia* also suggest that this trait is common in *Dokdonia* [49].

#### Genomic islands and lateral gene transfer (LGT)

Fig. 1C shows genomic islands in PRO95 and 4H-3-7-5 determined by calculating the correlation between local and global tetranucleotide frequencies along the genome, and Fig. 1D shows the phylogenetic affiliation of the foreign DNA. The XR-like gene sequence of PRO95 (see below) was located in close proximity to, but not inside of an identified genomic island. The correlation in this region (0.59) was, however, lower than the first quartile of correlations (0.64). LGT of single genes is very hard to detect [36], therefore the strong phylogenetic signal might be supportive enough although alterations in the oligonucleotide frequency do not clearly indicate LGT. Other than that, gene transfer in PRO95 seems to be limited, since the genomic islands were few and rather small. The acquired foreign DNA was in most cases derived from the phylum *Bacteroidetes*, with a frequency distribution that resembles the phylogenetic distance. 85 genes had their closest relatives in genera of the *Flavobacteriales* other than *Dokdonia.* Most of these genes might also have been lost in the other *Dokdonia* species, as there is no clear genomic signature indicating LGT. In addition, 38 genes showed similarity to other members of the *Flavobacteriia*. Only 22 genes were found to have been recruited from other bacterial phyla, i.e. *Firmicutes* (7) and *Proteobacteria* (15). For single genes acquired from these phylogenetic groups an oligonucleotide frequency signal was missing, too. Among the LGT genes are those for thiamine biosynthesis, heavy metal transporters and a sodium/hydrogen exchanger. Interestingly, PRO95 recruited a ribonuclease and DNA methylation genes from *Firmicutes* (Table S4).

#### A xanthorhodopsin-like protein is encoded in the genome of PRO95

A second predicted rhodopsin gene was found in the genome of *Dokdonia* sp. PRO95, encoding a protein of 280 amino acid residues (843 bp) which shows typical rhodopsin features. The amino acid residue Lys231 (EBAC31A08-numbering [50]) in helix G binds the cofactor retinal and is completely conserved in all PRs and XRs [4], [6], [23]. The amino acid residues functioning as proton acceptor and proton donor differ from those described previously. Instead of Asp97 (D) and Glu108 (E), the related amino acids Asn97 (N) and Gln108 (Q) are found. In comparison to Asp and Glu, these amino acids contain an amid side chain instead of a caboxyl group, indicating also a possibly new group of rhodopsins. The same amino acids at these positions were also found in rhodopsins of the strains 4H-3-7-5, *Gillisia limnaea* R-8282^T^ ( = DSM 15749) [51], [52], *Truepera radiovictrix* RQ-24^T^ ( = DSM 17093) [53], [54], *Citromicrobium bathyomarinum* JL354 [55], *Citromicrobium* sp. JLT1363 [56], *Fulvimarina pelagi* HTCC2106^T^ [57] and *Phycisphaera mikurensis* NBRC 102666^T^. At position 105, which is mainly responsible for rhodopsin spectral tuning, the amino acid residue Leu (L) was detected [50]. Without considering the possible influence of further amino acid residues [58] or the influence of the putative carotenoid antenna, which might shift the absorbance maximum, the second rhodopsin of PRO95 seems to be a green-light absorbing rhodopsin.

The retinal binding pocket shows similarities to that of described XRs. Four of twelve amino acid residues involved in chromophore binding are identical to those in XR of *Salinibacter ruber* [59]. Interestingly, the XR of *Gloeobacter violaceus* PCC 7421 [60] similarly shares only seven of the twelve amino acids involved in chromophore binding with *S. ruber* (Gly156, Thr160, Asn191, Leu197, Ile205, Tyr207, and Met211, amino acid residue numbering according to the XR sequence of *S. ruber*). When its XR gene is expressed in *E. coli* and reconstituted with both salinixanthin and retinal it behaves like the XR of *S. ruber* [61]. Moreover, the pigment of *G. violaceus*, echinenone, reconstitutes a functional XR molecule *in vivo* [8], indicating that different XRs may use different types of carotenoids as antenna molecules. Carotenoids belong to the most structurally diverse and widely distributed pigments in nature. In both chlorophyll and bacteriochlorophyll based photrophy, carotenoids serve as accessory pigments in light-harvesting complexes as well as photo-oxidative protectors [62]–[64]. The genomes of *Dokdonia* sp. PRO95 and *Dokdonia* sp. 4H-3-7-5 which both encode for PR and a XR-like protein, lack the *crtO* gene, suggesting that their XR might be associated to an antenna carotenoid different from salinixanthin.

#### Phylogenetic affiliation of the two rhodopsins of PRO95

**Figure 2:**
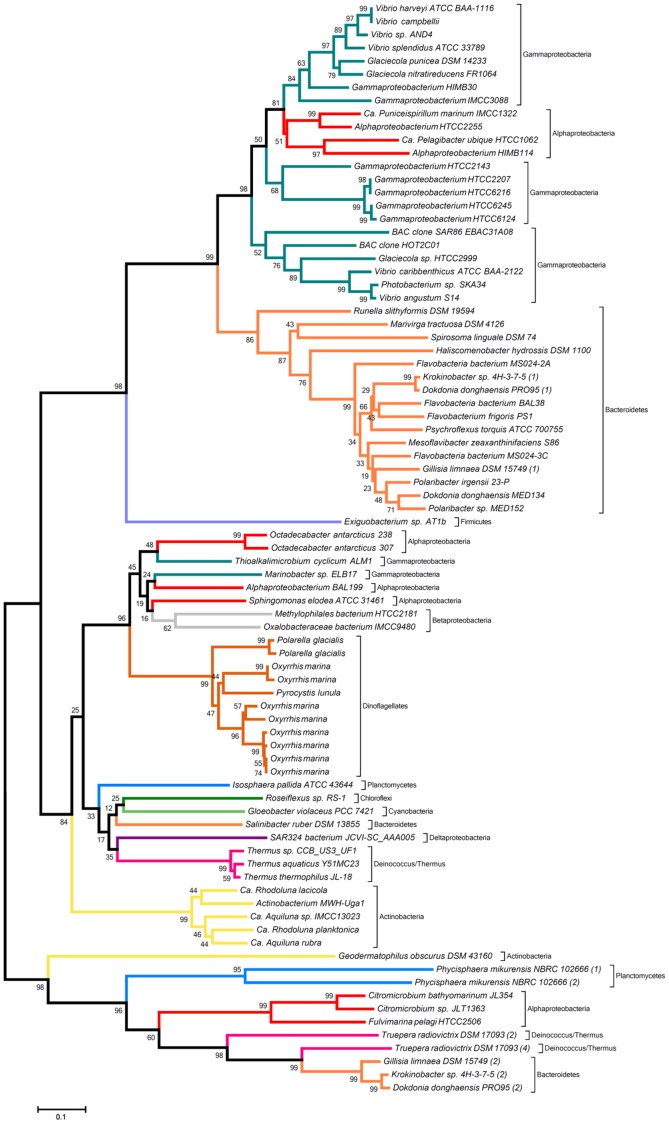
Neighbor-joining phylogenetic tree of the two rhodopsin protein sequences found in PRO95. The scale bar shows the number of amino acid substitutions per site. The colors of the tree branches as well as their legends indicate the phylum the different bacteria belong to. Accession numbers for the sequences used for the tree can be found in the Information S1.

The phylogenetic affiliation of the two rhodopsin protein sequences of PRO95 is shown in Fig. 2. The PR sequence is most closely related to sequences found within the *Bacteroidetes* phylum, especially in many representatives of the family *Flavobacteriaceae*. The PR-encoding protein sequence is 98% identical to one of the two rhodopsin sequences of both 4H-3-7-5 and R-8282^T^ and 73% to that of MED134. It shows further similarities to PR sequences found in *Alphaproteobacteria* and *Gammaproteobacteria*. The phylogenetic tree for PR is largely congruent with the 16S rRNA tree, indicated by coloring in Figure 2.

The second rhodopsin sequence found in the genome of PRO95 encodes an unclassified rhodopsin located in a cluster of XR and rhodopsin proteins related to XRs in a diverse range of phyla. A highly similar protein is present in a related *Dokdonia* sp. strain 4H-3-7-5 (98% identity). However, the next most closely related rhodopsin sequences were found in *Gillisia limnaea* R-8282^T^ (88% identity) [52] and in *Truepera radiovictrix* RQ-24^T^ [53], [54] (60% and 47% identity), which is a representative of the *Deinococcus/Thermus* phylum. Homologues of this second rhodopsin sequence (31% to 36% identity) were additionally found in a group of *Alphaproteobacteria* and *Planctomycetes*. These rhodopsins have until now only been analysed in *Salinibacter ruber* M31^T^ [65], [66] and *Gloeobacter violaceus* PCC 7421 [60], where they were shown to be XRs, i.e. proteorhodopsins with an additional antenna. The rhodopsins in *Citromicrobium bathyomarinum* JL354, and *Fulvimarina pelagi* HTCC2506^T^ have been annotated as XRs and are the only rhodopsins present in these bacteria [55], [57].

The strains *Dokdonia* sp. PRO95 and *Dokdonia* sp. 4H-3-7-5 were isolated from temperate marine environments (surface water layer and subseafloor sediments). *G. limnaea* R-8282^T^ was isolated from a microbial mat at Lake Fryxell (Antarctica) [51], [52], while *T. radiovictrix* RQ-24^T^ was isolated from a hot spring [53], [54]. Strain RQ-24^T^ is extremely resistant against radiation and grows optimally at a temperature of 50°C whereas the strains PRO95, 4H-3-7-5 and R-8282^T^ grow only up to less than 37°C. It is therefore unlikely that this rhodopsin, which shows homologies to known XR proteins, has been transferred to them in an extreme habitat. Organisms carrying XR-like homologues seem to be widespread and were found in diverse marine habitats, only some of which were extreme, e.g. sea-ice brine, crystallizer ponds of salterns, or hot springs (Table S2).

#### Genomic environment of both rhodopsin genes

**Figure 3:**
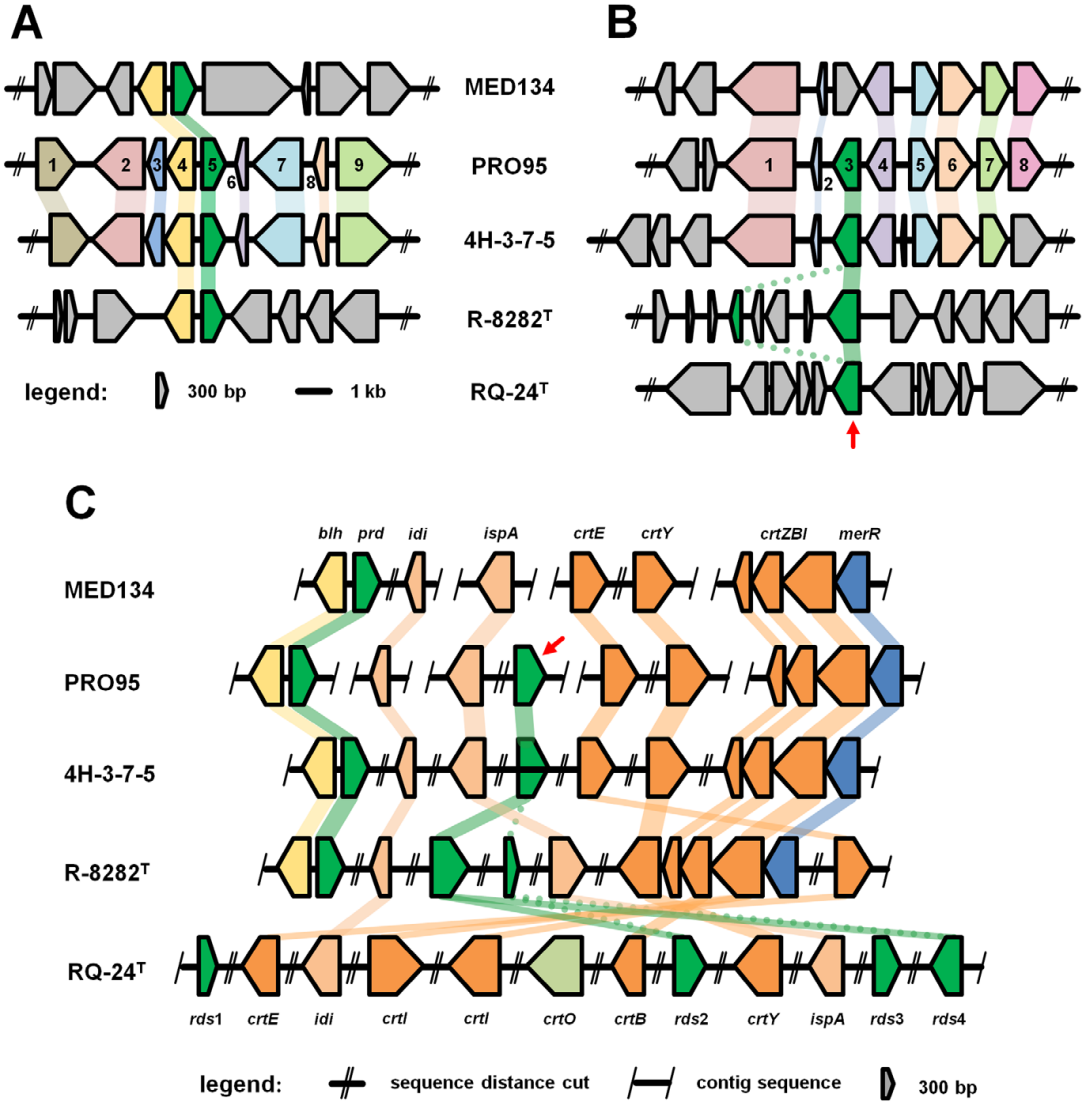
Genomic environment of the PR gene (A) and the XR gene (B), and a comparison of genes for carotenoid biosynthesis (C) in PRO95 and closely related strains. Genes were identified using NCBI Blast Analysis [43] and a comparison was performed with reference genomes. The lengths of ORFs and non-coding regions were manually retrieved. Similarities in genes are shown in same color and stripes. Non-related genes are shown in gray. The red arrow shows the position of the putative XR-gene. The Genbank accession numbers can be found in Information S2. **A.** 1 protein of unknown function; 2 peptidase M1 membrane alanine aminopeptidase; 3 hypothetical protein; 4 β-carotene 15,15-monooxigenase, brp/blh family (*blh*); 5 proteorhodopsin; 6 hypothetical protein; 7 FAD-dependent pyridine nucleotide-disulfide oxidoreductase; 8 membrane protein; 9 transcription termination factor Rho. **B.** 1 polyribonucleotide nucleotidyltransferase; 2 30S ribosomal protein S15; 3 putative xanthorhodopsin; 4 gliding motility protein GldA; 5 prephenate dehydratase; 6 aminotransferase; 7 prephenate dehydrogenase; 8 3-deoxy-phosphoheptunolate synthase. **C.** RH stands for the Deinococcus unclassified microbial rhodopsin gene.

The genomic environment of the PR gene (which is lacking in strain RQ-24^T^), is almost completely syntenic in the two closely related flavobacterial strains PRO95 and 4H-3-7-5. In contrast, strain MED134 and R-8282^T^ contain only the *blh* and PR gene, but none of the other genes found in this genomic region of PRO95 and 4H-3-7-5 (Fig. 3A). The genomic region around the XR-like rhodopsin gene of PRO95 is also highly syntenic to that of strain 4H-3-7-5, although these genes are not known to be related to the rhodopsin function itself (Fig. 3B). Interestingly, while R-8282^T^ and RQ-24^T^ contain the XR-like-encoding sequence but none of the surrounding genes, MED134 has nearly all the adjacent genes, but lacks the XR-like encoding sequence. Instead, it carries a hypothetical protein at this position. The data suggest that most likely only the XR-related gene has been taken up or lost by LGT, an event that seems to have occurred frequently in the marine habitat.

#### Genes for carotenoid and retinal biosynthesis

For the biosynthesis of β-carotene from isopentenyl diphosphate (IPP) six enzymes are required [23], [24]. β-carotene is a precursor for the biosynthesis of carotenoids and can be directly transformed into two molecules of retinal by the β-carotene 15,15′-monooxygenase encoded by the *blh*-gene. All these genes are present in the genomes of strain PRO95, MED134, 4H-3-7-5 as well as in strain R-8282^T^ (Fig. 3C). A rather complex situation can be observed in strain RQ-24^T^. Its genome encodes two *crt*I genes as well as a *crtO* gene, which is required for the formation of the carotenoid antenna of the XR. Genome analysis of PRO95, 4H-3-7-5 and R-8282^T^ revealed the lack of this *crtO* gene, indicating that those rhodopsins use another antenna protein or they function without an additional antenna, suggesting a new group of rhodopsins. Surprisingly in strain R-8282^T^ as well as RQ-24^T^ more than two rhodopsin genes were found: strain R-8282^T^ encodes 3 rhodopsin genes – one PR, one unclassified rhodopsin related to XR sequences and one truncated rhodopsin sequence. Strain RQ-24^T^ encodes even 4 rhodopsin genes. Two of them (*rds2* and *rds4*) are similar to the second rhodopsin of strain PRO95. The third one (*rds1*) appears to be truncated, and *rds3* seems to be a sensory rhodopsin (Fig. 3C).

#### Sigma factors

The primary sigma factor in bacteria is RpoD (σ70), which binds to conserved sequences of strong promoters and plays a critical role in regulating the expression of housekeeping genes. As reported earlier, MED134 encodes two RpoD homologs [48], and the same is true for PRO95 and 4H-3-7-5. One of the MED134 RpoD homologs (MED134_12871) is 93% identical to the RpoD of *Flavobacterium johnsoniae* UW101 for which the consensus promoter sequence is known and common in flavobacteria: TTGNTANNTTTG. In MED134, the consensus promoter sequence of RpoD is found upstream of 70 genes, including the PR gene. In PRO95 it is found upstream of 66 genes, but not upstream of genes related to light and energy conservation, i. e. the PR or XR-like genes. The second MED134 RpoD is 100% identical to the second RpoD in PRO95. But for this one the consensus promoter sequence is not known in flavobacteria. 4H-3-7-5 also has an RpoD that is 97% identical to the first MED134 RpoD and the second RpoD (MED134_05474) is also 100% identical to Krodi_2724. Like in PRO95 the consensus sequence of RpoD is not upstream of any of the opsin genes.

One might conclude that the opsins of PRO95 or 4H-3-7-5 may not function as housekeeping genes because the main sigma factor does not bind to them. This is however in contrast to the constitutive expression of PR in PRO95. Moreover, the second homologue of the main sigma factor, which is present in all three strains, and whose promotor sequence is not known, could also play a crucial role for the control of the rhodopsin genes. Thus further work is needed to elucidate the regulation of PR expression in these flavobacteria.

### Physiology

#### Phenotype microarrays

**Figure 4:**
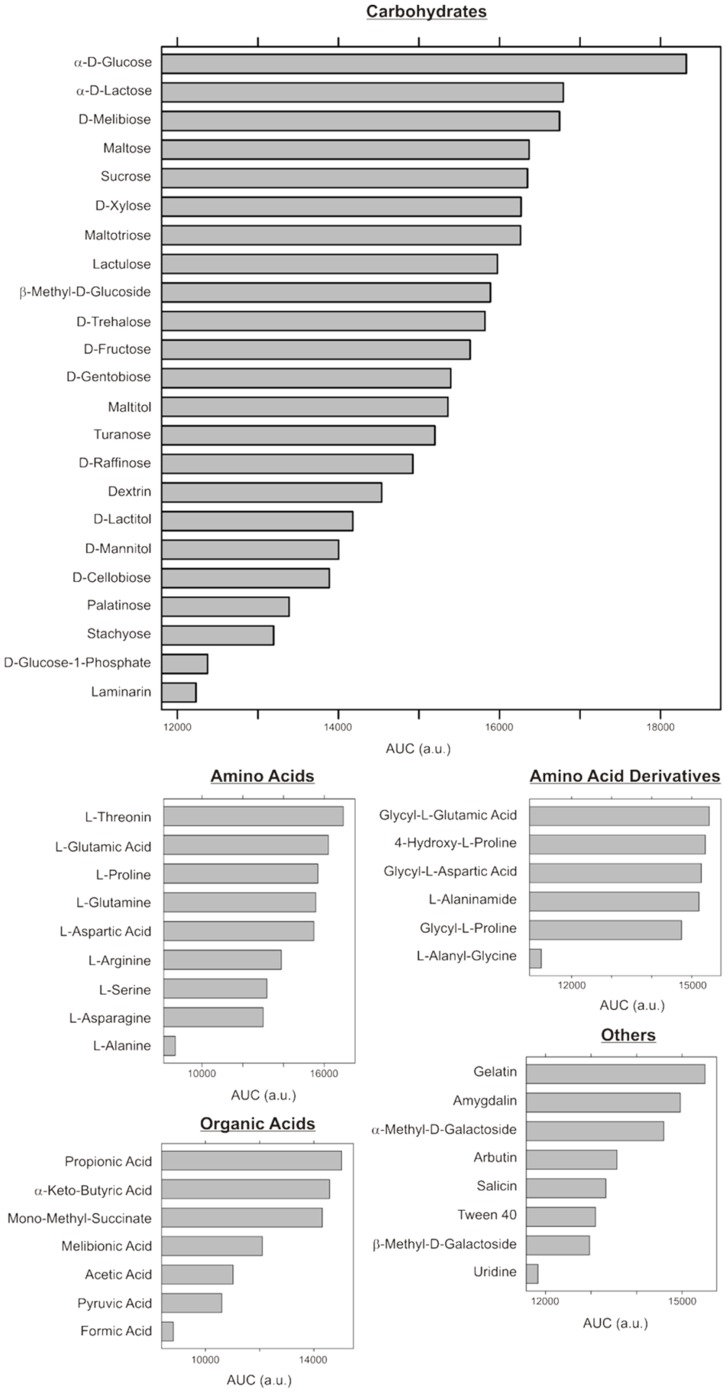
Carbon source utilization. The ability of PRO95 to take up and respire 190 different carbon sources was tested using the Omnilog system (plates PM1 and PM2). The area under curve (AUC) was determined as a measure of respiratory activity for each tested carbon source. Respiratory activity was assumed to be positive for an AUC that was 50% higher than that of the negative control. Two independent experiments were performed which showed the same results.

For characterization of carbon source metabolism in strain PRO95 we used the Omnilog system (Biolog platform). Phenotype microarray plates PM1 and PM2 were used. They contain 190 different carbon sources together with a mix of IF-0, artificial seawater medium, vitamins, trace elements, sodium bicarbonate buffer and DyeD. DyeD is a tretazolium redox dye which enables the detection of respiration by NADH-dependent reduction, which results in purple color. The formation of the purple color thus reflects the import as well as the metabolic conversion of a specific substrate, but not necessarily growth. Experiments with both PM plates were performed in two independent replicates which showed identical results. To allow quantitative comparison of results, the area under curve (AUC) was determined as a measure of respiratory activity for each tested carbon source using Simpson’s rule. Respiratory activity was assumed to be positive for an AUC that was 50% higher than that of the negative control. The data indicate that PRO95 can import and metabolize 53 different carbon sources. We identified 23 different carbohydrates, including glucose. In addition PRO95 metabolized 15 different amino acids (including L-glutamic acid) and amino acid derivatives (e.g. glycyl-L-glutamic acid), 7 different organic acids (e.g. propionic acid) and 8 compounds that were related to other carbon sources (e.g. gelatin) (Fig. 4).

#### Development of a defined cultivation medium

A defined cultivation medium is not known for any of the flavobacteria and would be a large step forward in understanding their physiology. It was not possible to develop a minimal medium for strain PRO95 containing only one carbon source together with a universal mix of vitamins (DSMZ medium 141) and trace elements (DSMZ medium 792).

As a further step, the genome was investigated for cultivation related information. Biosynthesis pathways for all 20 proteinogenic amino acids were found in the genome of PRO95 by inspection of the KEGG pathway maps. Thus PRO95 should not be auxotrophic for any of them. Inspection of the KEGG pathway maps revealed also the possible biosynthesis of non-proteinogenic amino acids like L-citrulline, L-ornithine and argininosuccinate, which play a major role within the urea cycle. PRO95 should also have the ability to synthesize D-alanine, D-glutamine, D-glutamate, D-phenylalanine and D-proline as well as β-alanine which is required for coenzyme A biosynthesis. Given the preference of PRO95 (and flavobacteria in general) for degradation of high molecular weight compounds, especially proteins, this is an unexpected finding. The cultivation approach was based on artificial seawater which was amended with Wolfés Mineral Elixier (DSMZ medium 792), NH_4_Cl, KH_2_PO_4_, Na_3_CO_3_, and Wolin-Vitamins (DSMZ medium 141). The pH of the medium was adjusted to pH 7.3–7.5, cultures were incubated at 28°C in weak light (40 Watt light bulb). The preculture was grown in marine broth 2216 medium filled into serum bottles under air atmosphere, and the main cultures were inoculated with 1% (v/v) of the preculture. The medium contained all minerals required by bacteria, N and P in large quantities, and the vitamins known to be required by enzymes, including thiamine and cobalamin. Therefore, theoretically supplementation of this medium with a carbon source should have been sufficient for growth of PRO95. Based on the carbon respiration data (Fig. 4) the carbon sources D-glucose and L-glutamate were chosen as substrates. However, no growth was obtained with glutamate (10 mM) or glucose (2 mM). When L-histidine or malate were supplied in small amounts to glutamate, weak growth was obtained after 3 days, whereas L-tryptophan, L-isoleucine, L-proline, benzoate or capronate had no stimulatory effect. Cultures grown in defined medium with glutamate did, however, not continue to grow when transferred to fresh medium. Using glucose as a carbon source and a mixture of amino acids as supplement, growth was much slower, reaching stationary phase in about three months, but this culture could be transferred to a similar medium and showed at least some weak growth after three months again. By contrast, growth and repeated subcultivation was possible in artificial seawater media with at least a supplement of 0.01% yeast extract.

The data suggest that an unknown component has been lacking in the defined media, which is normally present in yeast extract, and which might have a regulatory role, rather than act as a growth substrate. Yeast extract contains not only single amino acids, but also dipeptides, oligopeptides, and diketopiperazines, which can form during autoclaving [67], [68]. A possible role of diketopiperazines as quorum sensing modulators has been described [69]. In myxobacteria, quorum sensing-dependent growth regulation is performed by the so called “A-factor”, a diffusible cell-cell communication signal which is a mixture of amino acids and peptides [70]. No mechanism for density-dependent gene regulation has been described for flavobacteria so far.

#### Expression of the two rhodopsin genes and the β-carotene pathway in PRO95

For transcriptome analysis two samples were used: For the first one, *Dokdonia* sp. PRO95 was cultivated under natural light/dark regimes in half concentrated marine broth 2216 medium amended with sea salts resulting in a final carbon concentration of 121 mM as described previously [25]. The mRNA was enriched from isolated total RNA using Capture Oligonucleotides together with Oligo MagBeads (MicrobExpress Kit). For the second sample, PRO95 was incubated at a cell density of 10^6^ cells/ml in North Sea water amended with N and C as described [23]. Due to the low amount of cellular material, total RNA obtained from the low carbon sample had to be amplified prior to mRNA enrichment and sequencing.

The RNA was amplified using the MessageAmpII-Bacteria Kit and then rRNA was partly removed using the Terminator 5′ Phosphate-dependent Exonuclease. 155 and 56 out of 3372 firstly annotated ORFs could not be detected in the transcriptome from the low and high carbon cultivation sample, respectively. 40 ORFs could not be found in both datasets. In many cases those genes were located in close proximity on the genome, indicating that the lack of transcripts was likely due to inactivity of these genes and not due to a technical artifact. Both the scatter plot and the calculated Pearson correlation coefficient of 0.924 point to a linear relationship between both datasets suggesting that amplification of mRNA was linear and did not introduce a systematic bias when the obtained data are compared to non-amplified samples. Using a linear model approach, a slope of 0.8235 (standard error 0.0179, p-value = 0.0) was calculated, further supporting a linear relationship between both samples.

**Figure 5:**
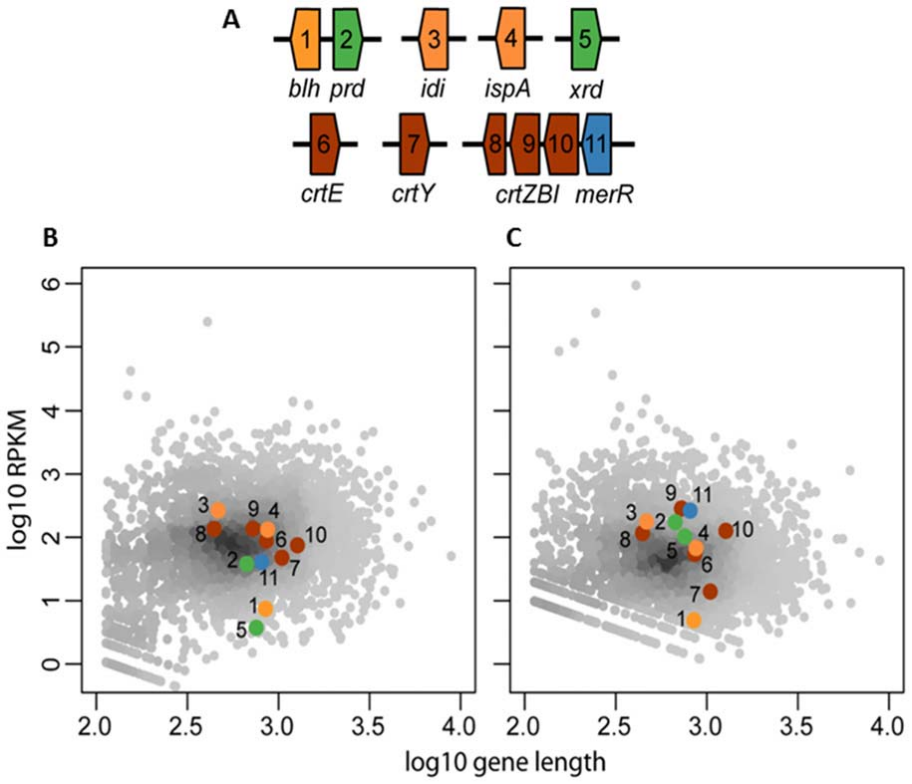
Expression of the two opsins and the retinal biosynthesis genes in PRO95 determined by RNAseq. The RPKM (10^9^*C/NL, where C is the number of reads mapped on the transcript, N is the total number of mapped reads and L is the length of the transcript) as a measure of expression is plotted against the gene length for a transcriptome analysis of *Dokdonia* sp. PRO95 in high carbon medium without mRNA amplification (A) and low carbon medium with mRNA-amplification (B). PR-related genes are highlighted and numbered according to Fig. 3.

RNA-sequencing revealed the expression of both rhodopsin genes and all genes necessary for retinal biosynthesis (Fig. 5). The transcriptome analyses also revealed that the *blh*-gene, encoding β-carotene 15,15′-monooxigenase, catalyzing the last step in retinal synthesis, was expressed weakly but detectably in both experiments. All further genes necessary for retinal biosynthesis (*idi*, *ispA*, *crtE*, *crtB*, *crtI*, *crtY*) were expressed on an average level except for *crtY* in seawater culture. Both genes catalyzing the two last steps of the retinal biosynthesis (*crtY* and *blh*) were weakly expressed.

These results were confirmed by RT-PCRs with cDNA produced from total RNA which revealed expression of both rhodopsin genes as well as the *blh*-gene (Figure S1).

#### Proton translocation measurements

**Figure 6:**
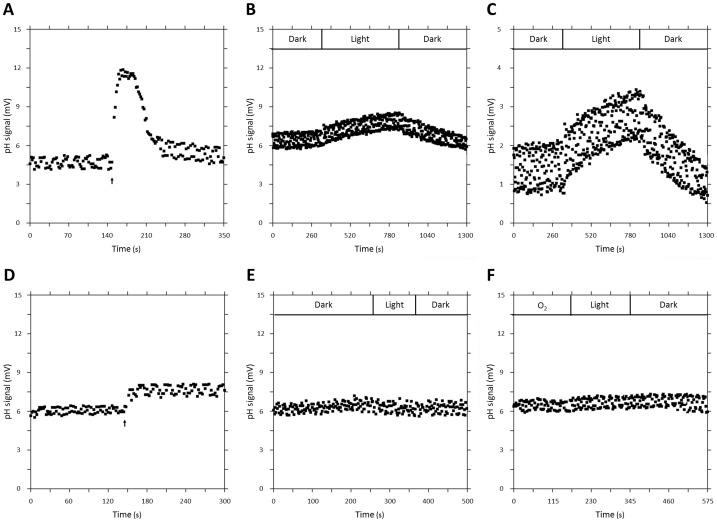
Oxygen-induced respiratory proton translocation and light-driven proton pump activity in suspensions of PRO95. (**A**) Proton translocation in response to an addition of oxygen-saturated media into the reaction chamber (black arrow). (**B**) Proton translocation in response to light. (**C**) Same as (B) at enlarged scale. (**D**) The system was calibrated with 50 nmol HCl, showing a distinct reaction directly after addition (black arrow). (**E**) The used medium without cells showed no response to light. (**F**) Cell suspensions treated with 25 µM 3,3′,4′,5-tetrachlorosalicylanilide showed no induction of proton translocation by oxygen or light.

For determination of oxygen-induced respiratory proton translocation as well as light-induced proton translocation in strain PRO95, washed cells were incubated in non-buffered salt solution under anoxic conditions. The addition of small amounts of oxygen-saturated artificial seawater medium led to a short-term decrease of the pH caused by the proton pumping activity of the respiratory electron transport chain (Fig. 6A). A period of proton excretion was followed by a period of proton uptake after oxygen exhaustion for a short period of time (Fig. 6A). It was most likely due to the uptake of protons via the ATP-synthase. To slow down the latter process, the chemical KSCN was used [71]. KSCN is a permeable anion that slows down the reflux of translocated protons by lowering the membrane potential [45]–[47], [72].

In addition to oxygen-induced respiratory proton translocation also a light-induced proton translocation was observed. In the light a distinct decrease in pH was detected, whereas an increase in pH, indicating proton uptake, was measured when light was switched off (Fig. 6B [low resolution] and 6C [high resolution]), indicating light-induced proton pumping and proton uptake via ATPase in the dark. For all proton translocation measurements several calibration steps and negative controls were implemented. As shown in figure 6D a calibration using 50 nmol HCl indicated the functioning of the measurement system. No reaction of media without any PRO95 cell suspension was detected in response to light (Fig. 6E). After addition of 25 µM of the uncoupler 3,3′,4′,5-tetrachlorosalicylanilide (TCS) to the cell suspension, neither oxygen-induced respiratory proton translocation nor light-induced proton pump activity wasdetected (Fig. 6F).

#### Growth of strain PRO95 in seawater media

**Figure 7:**
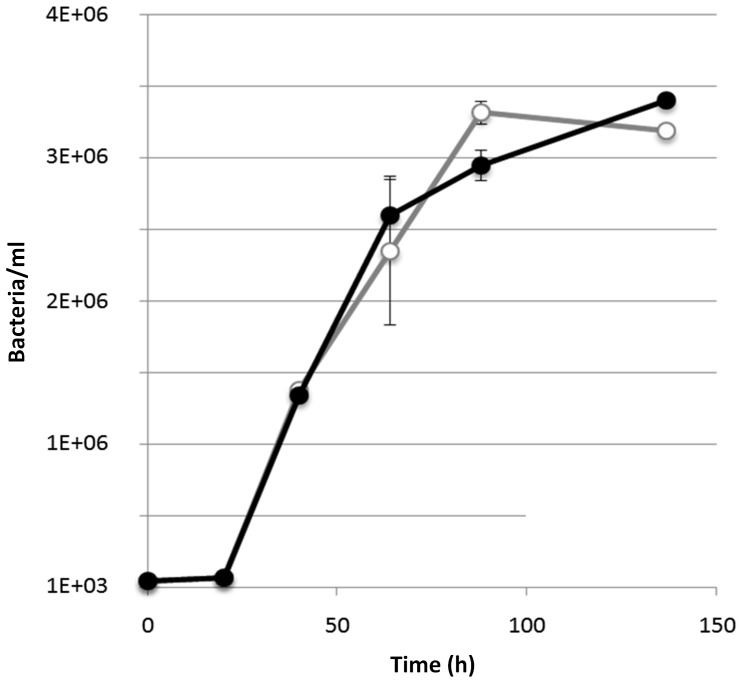
Growth of PRO95 in artificial seawater. PRO95 was cultivated in artificial seawater amended with 0.14 mM carbon both in the light and in the dark resulting in no growth advantage in the light (open circles) compared to the dark (filled circles).

To get insights into the physiological role of the two rhodopsin proteins encoded by PRO95 under starvation, its growth was investigated under oligotrophic conditions in artificial seawater amended with 0.14 mM carbon as described [23] under illumination and darkness. Cell counts revealed no growth advantage of strain PRO95 in the light compared to the dark (Fig. 7). Similar results were obtained in media containing autoclaved sterile filtered seawater amended with N and P (data not shown). These results are consistent with the growth data previously reported [25], showing no growth stimulation in the light in diluted marine broth 2216 media containing a carbon concentration down to 9.7 mM. No growth advantage under illumination in comparison to incubation in darkness was also observed for *Candidatus* Pelagibacter ubique [26], [30], *Polaribacter* sp. MED152 [27], *Vibrio* AND4 [28] and *Vibrio* BAA-1116 [29]. In contrast, a previous study showed higher cell yields and therefore a growth advantage of the PR-containing marine flavobacterium *Dokdonia* sp. MED134 in the light [23], [24], [48]. Seawater cultures starting with the same cell densities for both strains give cell yields 10-times higher for PRO95 as compared to MED134 ([23] and unpublished results). It thus seems that this bacterium can take better advantage of the nutrients present in seawater than MED134, regardless of being in the light or in the dark. Another way to study this might be to dilute seawater organic matter even further in the PRO95 culture. However, the goal of the experiment reported here was to compare the growth of the two similar strains under identical conditions.

## Discussion

Genome sequencing and analysis revealed the presence of a second bacterial rhodopsin gene in PRO95. It might represent a new type of rhodopsin and is similar to XR. This newly discovered rhodopsin gene is also present in related flavobacteria, e.g. *Dokdonia* sp. 4H-3-7-5 and *G. limnaea* R-8282^T^ [52] as well as in taxonomically diverse other bacterial groups suggesting that it has frequently been recruited through LGT. Its presence in phylogenetically distantly related groups indicates a “promiscuity” that may be connected to the extreme environments in which most of those rhodopsin-carrying organisms thrive. We show by RT-PCR and direct sequencing of mRNA that both rhodopsin genes as well as the complete pathway for the synthesis of the cofactor retinal are expressed by strain PRO95, suggesting an important role of rhodopsin-mediated phototrophy for this organism. A light-induced proton translocation activity was demonstrated, thus one or both rhodopsin proteins are functional. However, we detected no growth advantage of PRO95 when cultivated in seawater in the light compared to incubation in the dark. The flavobacterial PR has been expressed in *E. coli* and shown to be a proton pump. The new XR like protein found in PRO95 should definitely be investigated in an *E. coli* overexpression system to measure its response to light and its interaction with carotenoids.

Among *Haloarchaea*, multiple rhodopsins with different functions can exist in one single cell. For example, *Haloarcula marismortui*, a halophilic red pigmented haloarchaeon, encodes genes for six rhodopsins: two for BR, one for HR and three SRs [73], [74]. Additionally, in *Haloquadratum walsbyi*, a square shaped halophilic archaeon discovered first in a salt pool near the Red Sea, it could be shown that one rhodopsin shows both SRII and BR function [75].

In the domain *Bacteria* only a few cultivated strains are known encoding several genes for rhodopsins in one genome. An example is the extreme halophile *Salinibacter ruber* strain M31^T^ that belongs to the family *Rhodothermaceae* within the phylum *Bacteroidetes*. It was isolated from climax saltern chrystallyzers and its genome encodes for four different rhodopsin sequences (one bacterial XR, one archaeal HR and two archaeal SRs) [65]. The genus *Salinibacter* shares its extreme habitat with *Haloarchaea* thus it is thought that the archaeal rhodopsins were transferred into this halophilic bacterium by LGT due to the close association of these two organisms in hypersaline aquatic environments [65]. The scattered distribution of prokaryotic rhodopsin genes within *Bacteria* and *Archaea* has also been interpreted by other groups as a result of lateral gene transfer which switches between domains of life [65], [76], [77].

The amount of energy provided to strain PRO95 by PR (and possibly the XR like protein) apparently is too small to be detectable by cell counts or it is not converted into biomass in this organism Thus, much more sensitive parameters than cell number are required to unravel the function of rhodopsins in flavobacteria [30]. Experiments under starvation conditions are time consuming and contamination of the culture is a constant threat. Moreover, genetic systems for flavobacteria are very limited [78], [79]. Thus, the most precise experiment for an analysis of the global physiological role of PR, i.e. comparison of the wild-type with a gene deletion mutant, is not feasible. Comparing light and dark incubation is presently the only means to study the physiological role of PR.

However, although we are able to cultivate *Dokdonia* species in the laboratory, we are far from understanding their real metabolic requirements. This was demonstrated by our unsuccessful attempts to develop a defined cultivation medium for PRO95. In spite of the ability of PRO95 to metabolize a wide spectrum of carbon sources and to synthesize all proteinogenic amino acids, it failed to grow on cultivation media that provided a selection of compounds based on the current microbiological expertise, genome information, and carbon source respiration data. The striking differences observed when cultivating *Dokdonia* strains in artificial versus natural seawater (Gómez-Consarnau, unpublished results) also suggest that natural seawater may contain additional molecules which are required by the bacteria. Rather than being sources of carbon, nitrogen, or sulfur, the necessary cues might represent cell-cell communication signals or metabolic intermediates which must be provided by neighbouring cells in the natural habitat of *Dokdonia*. For example, *Candidatus* Pelagibacter ubique HTCC1062 is auxotrophic for certain vitamins [80] and thus critically dependent on the presence of bacteria that are able to excrete these vitamins. Although only tiny amounts are needed by the cells, concentrations in the ocean are far below these amounts in huge regions [81], [82].

## Supporting Information

Figure S1 **PCR amplifications.**
(DOCX)

Table S1 Summary of genomic DNA sequencing (A) and fosmid sequencing (B).
(DOCX)

Table S2 Overview of strains encoding rhodopsin homologues related to the second rhodopsin in PRO95.
(DOCX)

Table S3 Primers used in this study.
(DOCX)

Table S4 Genes acquired by lateral gene transfer.
(XLSX)

Information S1 Accession numbers of rhodopsin protein sequences in Figure 2.
(DOCX)

Information S2 Accession numbers of sequences in Figure 3.
(DOCX)
