# Supplementary material for: Genomics and Physiology of a Marine Flavobacterium Encoding a Proteorhodopsin and a Xanthorhodopsin-Like Protein
Source: PLoS One. 2013 Mar 4;8(3):e57487. doi: 10.1371/journal.pone.0057487 (PMC3587595; doi:10.1371/journal.pone.0057487)
Supplement: Figure S1 — PCR amplifications. (DOCX) [file pone.0057487.s001.docx]

**Figure S1. PCR amplifications. (A)** Complete PR gene (R1) and XR-like gene (R2) from genomic DNA of PRO95 (m_1_ - GeneRuler Ladder Mix, Fermentas, St. Leon-Rot, Germany). **(B)** Simultaneous amplification of fragments of PR and XR from genomic DNA of PRO95. Two different primer sets as described above (lane a1, b1 and lane a2 and b2) were used. For a1 and a2, genomic DNA of PRO95 was used as template, b1 and b2 shows the negative controls (m_1_ - GeneRuler Ladder Mix, Fermentas, St. Leon-Rot, Germany). **(C)** RT-PCR of PR and XR-like gene fragments showing simultaneous expression of both rhodopsin genes in PRO95 (+RT cDNA template, -RT RNA template without using reverse transcriptase to exclude genomic contamination, H_2_O negative control, m_2_ – GeneRuler 100bp DNA Ladder, Fermentas, St. Leon-Rot, Germany). **(D)** RT-PCR of a *blh*-gene fragment showing also expression under the described conditions. Abbreviations as in (C).

**Figure S1**

**
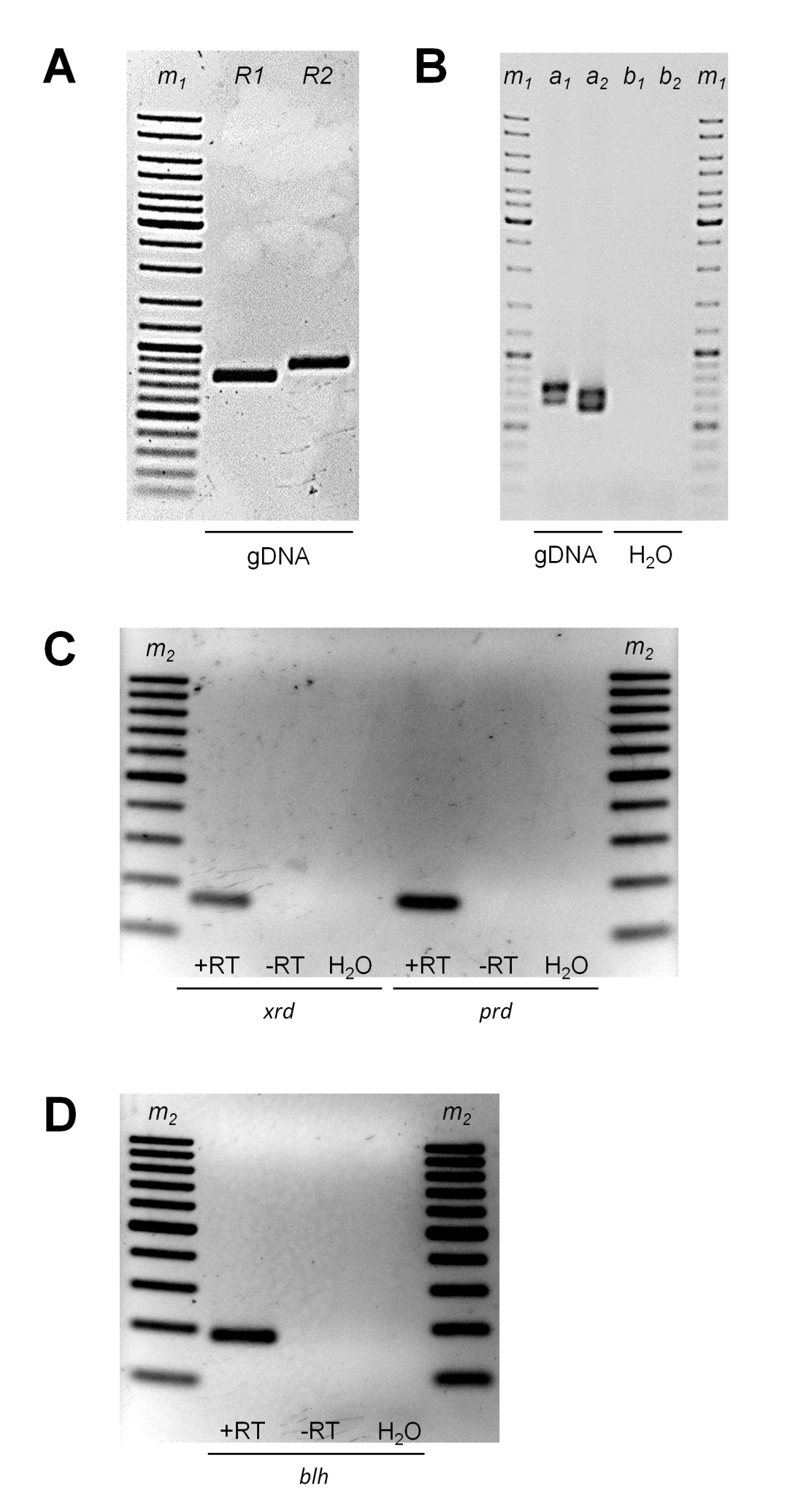
**
