# Supplementary material for: Genomics and Physiology of a Marine Flavobacterium Encoding a Proteorhodopsin and a Xanthorhodopsin-Like Protein
Source: PLoS One. 2013 Mar 4;8(3):e57487. doi: 10.1371/journal.pone.0057487 (PMC3587595; doi:10.1371/journal.pone.0057487)
Supplement: Table S1 — Summary of genomic DNA sequencing (A) and fosmid sequencing (B). (DOCX) [file pone.0057487.s002.docx]

**Table S1. Summary of genomic DNA Sequencing (A) and Fosmid Sequencing (B).** Total Illumina raw reads: amount of all reads; Median coverage depth: median depth of coverage at all covered loci; Nodes: constructed nodes for further assembly; N50: mean value of the 50 longest contigs; Contig (max size): the longest assembled contig; Total genome size: calculated genome size using all generated contigs; Reads used: number of reads used for assembly; Output: assembly output.

**Table S1 (A)**

| PRO95 DNA sample 76 bp PE run | |
| --- | --- |
| Total Illumina raw reads | 25.875.107 |
| Median coverage depth | 120 |
| Nodes | 1.138 |
| N50 | 60.135 bp |
| Contig (max. size) | 249.179 bp |
| Total genome size | 3345334 bp |
| Reads used | 21.870.804 (84.5%) |
| Output | 268 contigs / 131 scaffolds |

**Table S1 (B)**

| PRO95 Fosmid sample 150 bp PE run | |
| --- | --- |
| Total Illumina raw reads | 11.730.976 |
| Median coverage depth | 87.2 |
| Nodes | 227 |
| N50 | 61.218 bp |
| Contig (max. size) | 231.018 bp |
| Total genome size | 2.736.318 bp |
| Reads used | 8168843 of 8949218 (91.3%) |
| Output | 139 contigs |
