# Supplementary material for: Genomics and Physiology of a Marine Flavobacterium Encoding a Proteorhodopsin and a Xanthorhodopsin-Like Protein
Source: PLoS One. 2013 Mar 4;8(3):e57487. doi: 10.1371/journal.pone.0057487 (PMC3587595; doi:10.1371/journal.pone.0057487)
Supplement: Table S2 — Overview of strains encoding rhodopsin homologues related to the second rhodopsin in PRO95. (DOCX) [file pone.0057487.s003.docx]

**Table S2. Overview of strains encoding rhodopsin homologues related to the second rhodopsin in PRO95.**

**References**

Albuquerque L, Simoes C, Nobre MF, Pino NM, Battista JR, et al.(2005) *Truepera radiovictrix* gen. nov., sp. nov., a new radiation resistant species and the proposal of *Trueperaceae* fam. nov. FEMS Microbiol Lett 247: 161-169.

Anton J, Oren A, Benlloch S, Rodriguez-Valera F, Amann R, et al. (2002) *Salinibacter ruber* gen. nov., sp. nov., a novel, extremely halophilic member of the Bacteria from saltern crystallizer ponds. Int J Syst Evol Microbiol 52: 485-491.

Fukunaga Y, Kurahashi M, Sakiyama Y, Ohuchi M, Yakota A, et al. (2009) *Phycisphaera mikurensis* gen. nov., sp. nov., isolated from a marine alga, and proposal of *Phycisphaeraceae* fam. nov., *Phycisphaerales* ord. nov. and *Phycisphaerae* classis nov. in the phylum *Planctomycetes*. J Gen Appl Microbiol 2009, 55: 267-275.

Gai Z, Wang X, Zhang X, Su F, Wang X, et al. (2011) Genome Sequence of *Sphingomonas elodea* ATCC 31461, a Highly Productive Industrial Strain of Gellan Gum. J Bacteriol 193: 7015-7016.

Giovannoni SJ, Godchaux W, Schabtach E, and Castenholz RW (1987) Cell wall and lipid composition of *Isosphaera pallida*, a budding eubacterium from hot springs. J. Bacteriol 169: 2702-2707.

Gosink JJ, Herwig RP, and Staley RT (1997) *Octadecabacter arcticus* gen. nov., sp. nov., and *O. antarcticus*, sp. nov., nonpigmented, psychrophilic gas vacuolate bacteria from polar sea ice and water. Syst Appl Microbiol 20: 356-365.

Hahn MW (2009) Description of seven candidate species affiliated with the phylum *Actinobacteria*, representing planktonic freshwater bacteria. Int J Syst Evol Microbiol 59: 112-117.

Ivanova N, Rohde C, Munk C, Nolan M, Lucas S, Del Rio TG et al. (2011) Complete genome sequence of *Truepera radiovictrix* type strain (RQ-24). Stand Genomic Sci 4: 91-99.

Jiao N, Zhang R, and Zheng Q (2010) Coexistence of two different photosynthetic operons in *Citromicrobium bathyomarinum* JL354 as revealed by whole-genome sequencing. J Bacteriol 192: 1169-1170.

Kang I, Oh HM, Lim SI, Ferriera S, Giovannoni SJ, and Cho JC (2010) Genome sequence of *Fulvimarina pelagi* HTCC2506T, a Mn(II)-oxidizing alphaproteobacterium possessing an aerobic anoxygenic photosynthetic gene cluster and Xanthorhodopsin. J Bacteriol 192: 4798-4799.

Kang I, Lee K, Yang SJ, Choi A, Kang D, Lee YK, et al. (2012) Genome sequence of "Candidatus Aquiluna" sp. strain IMCC13023, a marine member of the *Actinobacteria* isolated from an arctic fjord. J Bacteriol 194: 3550-3551.

Klippel B, Lochner A, Bruce DC, Walston DK, Detter C, Goodwin LA et al. (2011) Complete Genome Sequences of *Krokinobacter* sp. Strain 4H-3-7-5 and *Lacinutrix* sp. Strain 5H-3-7-4, Polysaccharide-Degrading Members of the Family *Flavobacteriaceae*. J Bacteriol 193: 4545-4546.

Montresor M, Procaccini G, and Stoecker DK (1999) *Polarella glacialis*, gen. nov., sp. nov. (*Dinophyceae*): *Suessiaceae* are still alive! Journal of Phycology 35: 186-197.

Nakamura Y, Kaneko T, Sato S, Mimuro M, Miyashita H, Tsuchiya T et al. (2003) Complete genome structure of *Gloeobacter violaceus* PCC 7421, a cyanobacterium that lacks thylakoids. DNA Res 10: 137-145.

Oh HM, Lee K, Jang Y, Kang I, Kim HJ, Kang TW et al. (2011) Genome sequence of strain IMCC9480, a xanthorhodopsin-bearing betaproteobacterium isolated from the Arctic Ocean. J Bacteriol 193: 3421.

Okamoto OK. and Hastings JW (2003) Genome-wide analysis of redox-regulated genes in a dinoflagellate. Gene 321: 73-81.

Riedel T, Tomasch J, Buchholz I, Jacobs J, Kollenberg M, et al. (2010) Constitutive expression of the proteorhodopsin gene by a flavobacterium strain representative of the proteorhodopsin-producing microbial community in the North Sea. Appl. Environ. Microbiol. 76: 3187-3197.

Sharma AK, Sommerfeld K, Bullerjahn GS, Matteson AR, Wilhelm SW, et al. (2009) Actinorhodopsin genes discovered in diverse freshwater habitats and among cultivated freshwater Actinobacteria. ISME J 3: 726-737.

Sharma AK, Zhaxybayeva O, Papke RT, and Doolittle WF (2008) Actinorhodopsins: proteorhodopsin-like gene sequences found predominantly in non-marine environments. Environ Microbiol 10: 1039-1056.

Slamovits CH, Okamoto N, Burri L, James ER, and Keeling PJ (2011) A bacterial proteorhodopsin proton pump in marine eukaryotes. Nat Commun 2: 183.

Sorokin DY, Gorlenko VM, Tourova TP, Tsapin AI, Nealson KH, et al. (2002) *Thioalkalimicrobium cyclicum* sp. nov. and *Thioalkalivibrio jannaschii* sp. nov., novel species of haloalkaliphilic, obligately chemolithoautotrophic sulfur-oxidizing bacteria from hypersaline alkaline Mono Lake (California). Int J Syst Evol Microbiol 52: 913-920

Teh SB, Abdul Rahman AY, Saito YA, Hou S, Alam M (2012) Complete genome sequence of the thermophilic bacterium *Thermus* sp. strain CCB_US3_UF1. J Bacteriol 194: 1240.

Van Trappen S, Vandecandelaere I, Mergaert I, Swings J (2004) *Gillisia limnaea* gen. nov., sp. nov., a new member of the family *Flavobacteriaceae* isolated from a microbial mat in Lake Fryxell, Antarctica. Int J Syst Evol Microbiol 54: 445-448.

Ward BB and Priscu JC (1997) Detection and characterization of denitrifying bacteria from a permantly ice-covered Antarctic lake. Hydrobiologia 347: 57-68.

Yoon JH, Kang SJ, Park S, and Oh TK (2011) Reclassification of the three *Krokinobacter* species into the genus *Dokdonia* as *Dokdonia genika* comb. nov., *Dokdonia diaphora* comb. nov. and *Dokdonia eikasta* comb. nov. and emended description of the genus *Dokdonia* Yoon et al. 2005. Int J Syst Evol Microbiol 62:1896-1901.

Zheng Q, Zhang R, and Jiao N (2011) Genome sequence of *Citromicrobium* strain JLT1363, isolated from the South China Sea. J Bacteriol 193: 2074-2075.
