# Supplementary material for: Genomics and Physiology of a Marine Flavobacterium Encoding a Proteorhodopsin and a Xanthorhodopsin-Like Protein
Source: PLoS One. 2013 Mar 4;8(3):e57487. doi: 10.1371/journal.pone.0057487 (PMC3587595; doi:10.1371/journal.pone.0057487)
Supplement: Table S3 — Primers used in this study. (DOCX) [file pone.0057487.s004.docx]

**Table S3. Primers used in this study.**
