# Supplementary material for: Genomics and Physiology of a Marine Flavobacterium Encoding a Proteorhodopsin and a Xanthorhodopsin-Like Protein
Source: PLoS One. 2013 Mar 4;8(3):e57487. doi: 10.1371/journal.pone.0057487 (PMC3587595; doi:10.1371/journal.pone.0057487)
Supplement: Information S1 — Accession numbers of rhodopsin protein sequences in Figure 2. (DOCX) [file pone.0057487.s006.docx]

**Information S1: Accession numbers of rhodopsin protein sequences in Figure 2:** The phylogenetic tree contains the following sequences: *Vibrio harveyi* ATCC BAA-1116 (GenBank accession number YP_001445352), *Vibrio campellii* (ADC84422) *Vibrio* sp. AND4 (ZP_02194911), *Vibrio splendicus* ATCC 33789 (EGU39647), *Glaciecola punicea* DSM 14233 (ZP_09921023), *Glaciecola nitratireducens* FR1064 (YP_004870549), Gammaproteobacterium HIMB30 (ZP_09993982) Gammaproteobacterium IMCC3088 (ZP_08271926), *Candidatus* Puniceispirillum marinum IMCC1322 (YP_003552453), Alphaproteobacterium HTCC2255 (ZP_01447408), *Candidatus* Pelagibacter ubique HTCC1062 (YP_266049), Alphaproteobacterium HIMB114 (ZP_06055256), Gammaproteobacterium HTCC2143 (ZP_01616930), Gammaproteobacterium HTCC2207 (ZP_01223638), Gammaproteobacterium HTCC6216 (ABO88139), Gammaproteobacterium HTCC6245 (ABO88137), Gammaproteobacterium HTCC6124 (ABO88138), BAC clone SAR86 EBAC31A08 (AAG10475), BAC clone HOT2C01 (AAR05342), *Glaciecola* sp. HTCC2999 (ZP_03560325), *Vibrio caribbenthicus* ATCC BAA-2122 (ZP_07741624), *Photobacterium* sp. SKA34 (ZP_01161099), *Vibrio angustum* S14 (ZP_01236264), *Runella slithyformis* DSM 19594 (YP_004653826), *Marivirga tractuosa* DSM 4126 (YP_004054869), *Spirosoma linguale* DSM 74 (YP_003386489), *Haliscomenobacter hydrossis* DSM 1100 (YP_004446506), Flavobacteria bacterium MS024-2A (ZP_03702117), *Krokinobacter* sp. 4H-3-7-5 (1) (YP_004431469*), Dokdonia donghaensis* PRO95 (1) (ACM89772), Flavobacteria bacterium BAL38 (ZP_01734914), *Flavobacterium frigoris* PS1 (ZP_09895557), *Psychroflexus torquis* ATCC 700755 (ZP_01253360), *Mesoflavibacter zeaxanthinifaciens* S86 (ZP_09501337), Flavobacteria bacterium MS024-3C (ZP_03701022), *Gillisea limnaea* DSM 15749 (1) (ZP_09667301), *Polaribacter irgensii* 23-P (ZP_01117885), *Dokdonia donghaensis* MED134 (ZP_01049273), *Polaribacter* sp. MED152 (ZP_05108337), *Exigobacterium* sp. AT1b (YP_002885111), *Octadecabacter antarcticus* 238 (ZP_05063020), *Octadecabacter antarcticus* 307 (EDY76405), *Thioalkalimicrobium cyclicum* ALM1 (YP_004537746), *Marinobacter* sp. ELB17 (ZP_01737880), Alphaproteobacterium BAL199 (ZP_02189379), *Spingobacter elodea* ATCC 31461 (ZP_09955974), Methylophilales bacterium HTCC2181 (ZP_01551538), Oxalobacteraceae bacterium IMCC9480 (ZP_08273891), *Polarella glacialis* (AEF32711), *Polarella glacialis* (AEF32712), *Oxyrrhis marina* (ABV22427), *Oxyrrhis marina* (ADY17806), *Pyrocystis lunula* (AAO14677), *Oxyrrhis marina* (ADY17811), *Oxyrrhis marina* (ADY17808), *Oxyrrhis marina* (ABV22430), *Oxyrrhis marina* (ABV22432), *Oxyrrhis marina* (ABV22426), *Oxyrrhis marina* (ADY17809), *Isosphaera pallida* ATCC 43644 (YP_004180611), *Roseiflexus* sp. RS-1 (YP_001277280), *Gloeobacter violaceus* PCC 7421 (NP_923144), *Salinibacter ruber* DSM 13855 (YP_445623), SAR324 cluster bacterium JCVI-SC AAA005 (ZP_09914088), *Thermus sp.* CCB_US3_UF1 (YP_005654147), *Thermus aquaticus* Y51MC23 (ZP_03495873), *Thermus thermophilus* JL-18 (YP_006059019), *Candidatus* Rhodoluna lacicola (ACN42851), Actinobacterium MWH-Uga1 (ACN42848), *Candidatus* Aquiluna sp. IMCC13023 (ZP_09962421), *Candidatus* Rhodoluna planktonica (ACN42849), *Candidatus* Aquiluna rubra (ACN42850), *Geodermatophilus obscurus* DSM 43160 (YP_003410608), *Phycisphaera mikurensis* NBRC 102666 (1) (YP_005446284), *Phycisphaera mikurensis* NBRC 102666 (2) (YP_005444505), *Citromicrobium bathyomarinum* JL35 (ZP_06860850), *Citromicrobium* sp. JLT1363 (ZP_08702831), *Fulvimarina pelagi* HTCC2506 (ZP_01440547), *Truepera radiovictrix* DSM 17093 (2) (YP_003705905), *Truepera radiovictrix* DSM 17093 (4) (YP_003706581), *Gillisia limnaea* DSM 15749 (2) (ZP_09669334), *Krokinobacter* sp. 4H-3-7-5 (2) (YP_004429763), *Dokdonia donghaensis* PRO95 (2) (AEX55013).
