# Supplementary material for: Genomics and Physiology of a Marine Flavobacterium Encoding a Proteorhodopsin and a Xanthorhodopsin-Like Protein
Source: PLoS One. 2013 Mar 4;8(3):e57487. doi: 10.1371/journal.pone.0057487 (PMC3587595; doi:10.1371/journal.pone.0057487)
Supplement: Information S2 — Accession numbers of sequences in Figure 3. (DOCX) [file pone.0057487.s007.docx]

**Information S2: Accession Numbers of Sequences in Figure 3:** The alignment in A is based on the following accession numbers (from left to right): *Dokdonia sp.* MED134: ZP_01049269, ZP_01049270, ZP_01049271, ZP_01049272, ZP_01049273, ZP_01049274, ZP_01049275, ZP_01049276, EAQ40511; *Dokdonia* sp. PRO95: JN827388, JN827389, JN827390, JN827391, ACM89772, JN827392 , JN827393, JN827394, JN827395; *Dokdonia* sp. 4H-3-7-5: YP_004431465, YP_004431466, YP_004431467, YP_004431468, YP_004431469, YP_004431470, YP_004431471, YP_004431472, YP_004431473.

The alignment in B is based on the following accession numbers (from left to right): *Dokdonia* sp. MED134: ZP_01050266, ZP_01050267, ZP_01050268, ZP_01050269, ZP_01050270, ZP_01050271, ZP_01050272, ZP_01050273, ZP_01050274, ZP_01050275; *Dokdonia* sp. PRO95: JN827396, JN827397, JN827398, JN827399, JN827400, JN827401, JN827402, JN827403, JN827404, JN827405; *Dokdonia* sp. 4H-3-7-5: YP_004429768, YP_004429767, YP_004429766, YP_004429765, YP_004429764, YP_004429763, YP_004429762, YP_004429761, YP_004429760, YP_004429759, YP_004429758, YP_004429757; *Gillisia limnaea* R-8282^T^: relevant genes extracted from PRJNA82719, PRJNA50579; *Truepera radiovictrix* RQ24^T^ (DSM 17093): pseudo gene, YP_003706577, YP_003706578, YP_003706579, YP_003706580, YP_003706581, YP_003706582, YP_003706583, YP_003706584, YP_003706585, YP_003706586.

The alignment in C is based on the following accession numbers (from left to right): *Dokdonia* sp. MED134: ZP_01049272, ZP_01049273, ZP_01049324, ZP_01050290; ZP_01049338; ZP_08088224; ZP_01051268, ZP_01051269, ZP_01051270, ZP_01051271; *Dokdonia* sp. PRO95: JN827391, ACM89772, JN827406, JN827407, JN827400; JN827408, JN827409, JN827410, JN827411, JN827412, JN827413; *Dokdonia* sp. 4H3-7-5: YP_004431468, YP_004431469, YP_004429368, YP_004429742, YP_004429763, YP_004430061, YP_004430281, YP_004431196, YP_004431197, YP_004431198, YP_004431199; *Gillisia limnaea* R-8282^T^: relevant genes extracted from PRJNA82719, PRJNA50579; *Truepera radiovictrix* RQ-24^T^: YP_03703856, YP_003704782, YP_003705824, YP_003705847, YP_003705894, YP_003705897, YP_003705901, YP_003705905, YP_003706080, YP_003706284, YP_003706549, YP_003706581.
